# Management of chronic knee pain: A survey of patient preferences and treatment received

**DOI:** 10.1186/1471-2474-9-123

**Published:** 2008-09-18

**Authors:** Helene L Mitchell, Michael V Hurley

**Affiliations:** 1 De Montfort University, Leicester: Division of Psychology, Faculty of Health & Life Sciences, De Montfort University, Leicester, LE1 9BH, UK; 2 Rehabilitation Research Unit (King's College London), Dulwich Community Hospital, London, UK

## Abstract

**Background:**

A range of interventions exist for the management of knee pain, but patient preferences for treatment are not clear. In this study the management received by people with chronic knee pain, their management preferences and reasons for these preferences were recorded.

**Methods:**

At baseline assessment of a clinical trial of rehabilitation for chronic knee pain, 415 participants were asked about their i) previous management, ii) preferred treatment, if any, iii) whether they would undergo knee surgery and iv) reasons for their preferences.

**Results:**

*Previous management *– Medication was the most common treatment, followed by physiotherapy, 39 participants had received no treatment. *Preferences *– 166 patients expressed no treatment preference. Of those who expressed a preference the most popular option was physiotherapy, whilst *not *having surgery was the third most frequent response. The most common reason for preferring physiotherapy and not wanting surgery was prior experience.

*Willingness to accept surgery *– 390 participants were not waiting for knee replacement surgery, and overall 81% would not accept surgery if offered, usually because pain was not perceived to be severe enough to warrant surgery.

**Conclusion:**

Most chronic knee pain is managed with medication despite concerns about safety, efficacy and cost, management guidelines recommendations and people's management preferences. Previous experience and perceptions of need were major determinants of people's preferences, but many people were unaware of management options. Appreciating patient preferences and provision of more information about management options are important in facilitating informed patient/clinician discussion and agreement.

**Trial Registration:**

Current Controlled Trials, ISRCTN 94658828

## Background

Disabling chronic knee pain is very common [1,2]. Although evidence-based guidelines recommend exercise, education, and medication [3], management of chronic knee pain usually involves palliative medication, in spite of its potential risks and costs [4,5]. Fewer people are referred to physiotherapy [6] and only people with severe, disabling pain are referred for total knee replacement (TKR) surgery [7].

There have been calls for management decision-making to shift from doctor-determined to one which includes greater patient involvement [8]. This requires healthcare professionals understanding peoples' health beliefs and preferences, and appreciating that interventions that conflict with these beliefs and preferences may lead to dissatisfaction with care and non-adherence [9]. It also requires that people are aware of management options and their consequences.

Studies of patient preferences provide valuable insights into the way individuals make decisions about treatment. When asked about pharmacological therapies, the most important deciding factor when choosing a treatment was the risk of adverse side-effects; people prefer less efficacious treatments that carry a lower risk of side-effects [10]. However, if a patient's understanding of pain or the action of a medication is inaccurate or incomplete, this can strongly influence their treatment preference [11]. When asked about surgery, Figaro et al [12] identified six themes that explained participants' decisions not to undergo surgery (preference for natural remedies, negative expectations of surgery, belief in God's control, preference for continuing in the current state, relationships with specialists, fear of surgery or death), which were reduced to one super-ordinate theme – patients "did not want to be cut" and were prepared to put up with pain rather than risk the possible complications of surgery.

Peoples' treatment preferences for the management of knee pain, and the rationale for these preferences have not been investigated. In this simple survey we documented the treatment received by 415 people with chronic knee pain, their treatment preferences and rationale for these preferences, and matched treatment received and preferences against the recommendations of the three most important clinical guidelines for management of knee pain/osteoarthritis [3,13,14].

## Methods

Participants were recruited from a randomised clinical trial (RCT) of rehabilitation for chronic knee pain. Broad inclusion criteria were adopted; participants had to be aged 50 years or older and have consulted a primary care physician for mild, moderate or severe knee pain of more than 6 months duration. Exclusion criteria were: lower limb arthroplasty; physiotherapy for knee pain in preceding 12 months; intra-articular injections in preceding 6 months; unstable medical conditions; inability/unwillingness to exercise; severe lack of mobility; inability to understand English. People were not excluded if they had stable co-morbidities common in this age group (e.g. type II diabetes, cardiovascular or respiratory disorders), back, lower or upper limb pain. The interventions compared were usual primary care and usual primary care plus individual or group rehabilitation. In total 418 patients were recruited from 53 GP practices in inner London, UK. Detailed description of the trial and intervention are available ([15]), but briefly, it was a pragmatic evaluation of a rehabilitation programme designed to improve self-reported function using exercise, education and self-management strategies to alter behaviour and dispel inappropriate health beliefs.

The study reported here is a simple overview of previous management and participants' treatment preferences in a relatively large cohort of people using a structured survey. During baseline assessment trial participants were all asked:

"*What treatment have you previously received for your knee, for instance have you ever had drugs, physiotherapy, surgery, osteopathy, acupuncture, or any other treatment?*"

"*Given the choice of any treatment – drugs, physiotherapy, surgery, osteopathy, acupuncture, any treatment at all – do you have a preference for one over another? Why?*"

"*Are you on a waiting list for a knee replacement?* (If response yes) *Will you accept it when offered?* (If response no) *would you accept one if you were offered in the future?*"

These or any other treatments participants reported they had received or preferred were noted on an assessment form, as well as interventions they *did not *want. No written notes or audios recordings were taken. There was no additional probing of patient's preferences.

Ethical approval was obtained from Local Research Ethics Committees of King's (Ref No. 99–261), St Thomas' and Guy's (Ref No. EC99/814) and Lewisham (Ref No. 00/04/09) Healthcare Trusts.

### Statistical Analysis

Descriptive statistics for previous treatment, preferences, and willingness to undergo knee replacement surgery were calculated. Since the duration of knee pain was not normally distributed and there was a large disparity in numbers of participants who reported receiving treatment (*n *= 380) and those who did not (*n *= 35) the non-parametric Mann-Whitney U-tests was used to establish if there were differences in demographic factors (age, disease duration) between people who had received treatment and those who had not.

## Results

Data were collected from 415 of the 418 trial participants. Not all participants responded to every question, consequently not every analysis contains the same number of participants. The mean age was 67 years (range 50–91); 203 (49%) were married, 97 (23%) were widowed; 275 (66%) were Caucasian, 87 (21%) were Afro-Caribbean; median duration of knee pain was 6 years (range 6 months – 60 years).

In Table 1 the treatments participants a) preferred, b) received are ranked in descending order, and a c) summary of the 3 principal clinical management guidelines which have been loosely categorised as first line (for people with early/mild pain causing little disability), second line (for more advanced/moderate pain and disability) and late (for severe pain and ability) interventions.

**Table 1:** Treatments a) preferred and b) received by participants ranked in descending order, with c) guideline recommendations.

**a) Participant preference (number)**	**Rank**	**b) Treatments received (number)^1^**	**c) Guideline recommendations**
(166) No preference stated	1	Drugs – analgesics or NSAIDs (343)	Non-pharmacological interventions *Education/information/advice on: Exercise; Footwear; Weight control; simple pain control heat/cold/TENS*
(102) Physiotherapy/Hydrotherapy	2	Physiotherapy/Hydrotherapy (169)	
(52) Not surgery	3	Surgery^2 ^(51)	
(27) Not drugs	4	No treatment (39)	
(19) Acupuncture	5	Acupuncture (35)	Pharmacological interventions *Simple analgesia – oral paracetamol, Topical agents*
(16) Surgery (including arthroplasty)	6	Steroid injections (24)	
(11) Drugs – analgesics or NSAIDs	7	Osteopathy (18)	
(6) Not acupuncture, osteopathy, physiotherapy	8	Alternative therapies (9)	
(5) Osteopathy	9	Homeopathic remedies (4)	Surgery *If unresponsive to all pharmacological and non-pharmacological interventions and still having significant impact*
(5) Homeopathic remedies	10	Joint aspiration (4)	
(2) Steroid injections	11		
(2) Alternative treatment	12		
(1) TENS	13		
(1) Weight loss	14		

^1^Some participants had received more than one form of "other" treatment.

^2^Includes arthroscopy, lavage, menisectomy, other minor procedures, there were no total knee replacements.

% – percentage

TENS – Transcutaneous Electrical Nerve Stimulation.

NSAIDs – Non-steroidal anti-inflammatory drugs

Treatment preferences (Table 1a). Many participants had no preference for treatment (n = 166/415, 40%), but only a small number (n = 14) could give a reason for this, which included: willing to try anything that might help (n = 3); uncertainty about best treatment option (n = 3); uncertainty about the actual problem (n = 2).

Of the participants who expressed a preference, the most popular option was physiotherapy (n = 102). Many reasons for choosing physiotherapy were offered (some participants provided more than one reason), the most frequent were; previously beneficial (n = 20); no experience with physiotherapy/exercise but it was thought it would be beneficial (n = 9); participants wanted to exercise (n = 6); non-invasive (n = 4); perceived to be a more acceptable alternative to medication (n = 4) or surgery (n = 3).

52/415 participants (13%) spontaneously volunteered they *did not *want surgery due to: negative experiences of surgery by themselves or others (n = 4); concerns about risks of surgery (n = 3); surgery perceived to be unnecessary (n = 3); concerns about post-operative pain (n = 1); considered themselves too old (n = 1). Sixteen participants stated a preference for surgery based on the positive experience of people they knew, or they held pessimistic attitudes about the prognosis of their condition and the ability of conservative intervention to relieve their problems.

Treatments received Table 1b. Some participants reported receiving more than one intervention. The majority of participants had received some form of treatment (n = 382/415 participants, 92%), mostly analgesics or non-steroidal anti-inflammatory drugs (NSAIDs) (n = 343, 83%), a large minority had received physiotherapy (n = 169, 41%). Some had received no treatment (n = 39, 9%).

There was no statistical difference in age between people who had received treatment and those who had not, but those who had been treated had experienced pain for longer (U = 5142, z = -2.02, p = 0.04).

**Figure 1:**
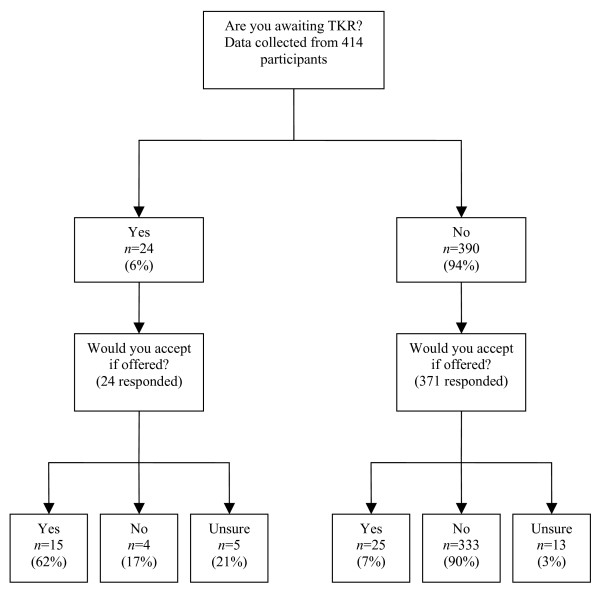
Outline of Patient Preferences for Surgery.

*Willingness for surgery *(Figure 1). Although 24 participants had been referred for orthopaedic assessment no definite decision to operate had been made, 9 participants said they would decline surgery if it was offered then. Of the 371 participants not on a waiting list for TKR who responded; 25 (7%) would consider TKR if offered; 13 (3%) were unsure; 333 (90%) were unwilling to undergo surgery. The most frequent reasons for not wanting surgery were: it was perceived to be unnecessary at present (n = 42); the idea of surgery was off-putting (n = 20); they had previously had, or knew of others who had had, bad experiences of surgery (n = 11); they were too young (n = 8); they were too old (n = 6); they had already had enough surgery (n = 5).

Participants awaiting surgery had longer duration of symptoms than those not on a waiting list (U = 2893.50, z = -2.72, p = 0.007).

## Discussion

This survey asked people from primary care with mild to moderate chronic knee pain about the treatment they had received and their treatment preferences, and matched these against the recommendations of evidence-based management guidelines. While most people had received some form of treatment, the treatment they received frequently did not reflect guideline recommendations or treatment preferences, though nearly a half of people expressed no treatment preference.

Clinical guidelines for the management of knee pain recommend initially employing non-pharmacological interventions (verbal and written information about the condition, self-management, physiotherapy, etc), supplemented with simple analgesia (paracetamol, topical agents), if necessary progressing to stronger second line oral analgesia (opioids and NSAIDs), reserving surgery for people unresponsive to conservative management [3,13,14]. In general, the clinical guidelines reflect lay people's treatment preferences for physiotherapy, not medication or surgery, and confirms the findings of previous studies [8,16]. However, in spite of management guidelines, the popularity and proven efficacy of physiotherapy [17,18], the unpopularity of medication, people's willingness to put up with pain to avoid taking medication [10] and serious concerns regarding the safety [19-21], efficacy [22] and costs [5] of medication, the majority of people had been prescribed analgesia or NSAIDs to alleviate their knee pain, while less than half had been referred to physiotherapy. Poor adherence to these clinical guidelines and the suboptimal management of osteoarthritis is not uncommon [23,24].

We did not specifically enquire what information, education or advice people had received, but most people will probably have received some information and advice about their condition informally during clinical consultations, rather than through a formal structured self-management programme. That no participant spontaneously mentioned information/advice as an intervention received suggests that they didn't receive any information, but if information was given people do not perceive it to be an intervention per se or it was ineffectual and people do not value its usefulness [16].

Lack of information about chronic joint pain, its causes, effects, prognosis and effective treatment options can have a major influence on people's preference for, acceptance of and adherence to treatment. Nearly half of the participants did not express any treatment preference. While some people will be happy to devolve decisions about their treatment to healthcare professionals [19], others will want to be involved in deciding their management. Coming to an informed decision about management requires that all available options are known. If the lack of treatment preference reflects limited awareness of effective treatment options, the ability of people to make informed decisions will be impeded. Tallon et al reported that although few people valued patient education/information many thought this should be a research priority, and the authors suggested it may be a way of people asking to take control of their condition [16]. If correct, not giving people information is denying them control over their condition. Our study suggests that despite the prioritisation of education/information recommended in clinical guidelines, if delivered at all, it is delivered ineffectually so people do not appreciate, utilise and implement the information. Ensuring people have sufficient information to make informed realistic decisions about treatment and eliciting treatment preferences may facilitate the decision-making process.

A sizable number of people (10%) had received surgery (arthroscopy, lavage, menisectomy) though there is little evidence these procedures are effective and they carry inherent risks. Although a few people had been referred for orthopaedic assessment they were undecided about accepting when given a date as they did not perceive it to be necessary [25]. This ambivalence highlights the unpopularity of surgery [26]; without prompting 10% of people stated they did not want surgery because of the nature of the intervention or the negative experiences of people they knew. It also highlights the disparity between lay people's perceptions about the need for medical/surgical intervention and a healthcare professional's assessment of need [25,27], emphasising the necessity of informed shared decision-making when deciding important management strategies. A small minority of people (<4%) nominated knee surgery as their preferred treatment.

This study has limitations that need to be considered. Firstly, it was not a "purpose-designed" in-depth survey of patient preferences, and it had to be carried out within the time and resource limitations of the RCT. We wanted to identify important issues that might be explored in greater detail, for example the nature of patient preferences, using in-depth interviews. Secondly, an RCT of a physiotherapy-based intervention might recruit people who prefer physiotherapy and are biased against medication, surgery and other interventions, while people with strong preferences against physiotherapy and exercise may have decided not to participate, giving a biased sample. However, the high proportion of people who had no treatment preference suggests selection bias did not influence the results greatly. Finally, information given during consent may have increased people's awareness of physiotherapy and other treatment options causing them to respond differently in the light of this knowledge. Again, if correct, more participants would have been expected to express treatment preferences.

In summary, we found a mismatch between people's treatment preferences, the treatment they had received and treatment that evidence-based guidelines recommend they should receive for chronic knee pain. Pressures on time and resources may be encouraging routine prescription of palliative medication [28], which is not recommended by current guidelines as an effective, efficient or safe way to manage chronic joint pain, instead of providing more and better information enabling people to make informed management choices. People in this study told us they wanted what the guidelines say they should get – initially non-pharmacological interventions with minimal medical/surgical intervention, with increasing medical/surgical intervention if these are ineffective. People's preferences and judgement of need for an intervention, their positive experience and concerns about outcome and side-effects influence acceptance of and adherence to treatment [29]. Enabling people to make informed choices, and delivering people's preferred healthcare interventions is likely to promote better adherence to optimal, effective management, especially when their preferences closely reflect the recommendations of the best available evidence.

## Competing interests

The authors declare that they have no competing interests.

## Authors' contributions

HM collected the data, performed the statistical analysis and prepared the manuscript. MH devised the study, collected the data and prepared the manuscript.

## Pre-publication history

The pre-publication history for this paper can be accessed here:


